# The mouth and oral health in the field of dementia

**DOI:** 10.1177/13634593211049891

**Published:** 2021-11-02

**Authors:** Sasha Scambler, Sarah Curtis, Jill Manthorpe, Kritika Samsi, Yvonne M Rooney, Jennifer E Gallagher

**Affiliations:** King’s College London, UK; King’s College London, UK; King’s College London, UK; King’s College London, UK; Community Special Care Dentistry, UK; King’s Dental Institute, UK; Teddington Community Dental Clinic, UK; Kingston Hospital, London, UK; King’s College London, UK

**Keywords:** chronic illness and disability, organisation of health services, theory

## Abstract

An ageing population, an estimated 47 million people currently living with dementia, and predictions of a threefold increase in people living with a diagnosis by 2050 have led the WHO to declare dementia a public health priority. Emerging research also suggests that dementia is linked to poor oral health and that oral health declines alongside cognitive decline. Drawing on Bourdieu’s concepts of field and capital, this paper presents an analysis of interview data from participants with dementia, carers and carer/diagnosed dyads participating in a qualitative study of the mouth and oral health. We argue that Bourdieu’s conceptual toolkit provides a way of contextualising experiences of oral health within dementia and un-picking the multi-layered impact of structure, institutions, biology, resource mobilisation and self in the context of a progressive disease which ultimately challenges knowledge of the self and the ability to interact with the world around us.

## Introduction

Dementia is often used as an umbrella term for a group of progressive conditions which affect the brain and impact on mental and cognitive processes. As many as 850,000 people in the United Kingdom (UK) are reported to be currently living with dementia, a number that is predicted to rise to 1 million by 2021, and dementia is a major cause of disability amongst older people (World Health Organisation, 2021). Global estimates suggest up to 47 million people are living with dementia, almost a third of whom live in low- or middle-income countries, with figures expected to increase three-fold by 2050. In response, the World Health Organisation (2017) declared dementia a public health priority. Dementia encompasses a range of progressive conditions (including Alzheimer’s disease, vascular dementia, frontotemporal dementia and dementia with Lewy bodies) which cause damage to nerve cells affecting messages sent to and from the brain. This results in memory problems, cognitive decline – particularly affecting the ability to locate oneself in time and space – and may affect communication.

There is a significant, and growing, body of research looking at the experiences of people living with dementia of interacting with health and care professionals (Adams and Gardiner, 2005; Brooker, 2003; Driessen and Ibáñez Martín, 2020; Prince et al., 2009; Samsi and Manthorpe, 2014). There are also a growing number of papers exploring the social relations of dementia (see e.g. the collection edited by Higgs and Gilleard (2017)). There remain, however, relatively few papers focusing on the interactions between dementia, oral health and oral health care. Of the research that does exist, it is suggested that people with dementia find it difficult to maintain oral health, therefore potentially experiencing diminished quality of life (British Dental Association, 2013; Kandelman et al., 2008). Furthermore, recent studies suggest a clear association between dementia and poor oral health (Daly et al., 2017) and that oral health declines as cognitive impairment increases (Manger et al., 2017).

A study by Lee et al. (2015) found that people living with dementia are less likely to visit a dentist regularly than other groups, and more likely to attend at later stages where symptoms are severe. Curtis et al. (2021) also found that oral health was only identified as a problem amongst community dwelling people living with dementia and their carers when a problem was identified, at which stage access to dental care was often challenging. Wider research further suggests that disabled people and other marginalised groups face numerous, ongoing barriers to accessing dental care (Borreani et al., 2008; British Dental Association, 2013; Chalmers, 2000; Scambler et al., 2015; Vanobbergen et al., 2007) which result in poorer oral health outcomes and the need for more extreme treatments. A lack of access and perceived need are compounded by the absence of clear guidance on the provision of oral health care for people living with dementia. NICE guidelines on ‘Oral Health for Adults in Care’ offer little in the way of guidance for people living in their own homes, stating only that people with dementia may be unable to communicate pain (National Institute of Clinical Excellence, 2018). Good oral health benefits older people in terms of dignity, social integration, nutrition and life without pain (Alzheimer’s Disease International, 2019) but research suggests that oral health is largely absent from ‘packages’ of dementia care.

A small number of papers have sought to apply aspects of Bourdieu’s theory of practice (Bourdieu, 1991, 1998) to dementia. This conceptual framework provides a means through which to explore the interplay between structure and agency, power and capital, giving an insight into the complexities of daily life in whichever sphere (or field) we choose to study. Bourdieu’s (1991, 1998) concepts of field and habitus seek to address the ways in which agency and reflexivity (habitus) are shaped by or embedded within structure (field). His conceptual toolkit was developed to help explain the practices that he observed within fields and to explore how and why people act as they do (Bourdieu, 1991). Rhynas (2005) focused on the potential of Bourdieu’s concepts in the field of dementia nursing care, suggesting that education, science, the media and the arts all play a part in shaping the structure of the field along with the organisational structure of the hospital system and of old age. She further suggested that capital accumulation and transaction are diminished by dementia. Other researchers have gone further and applied Bourdieu’s concepts of field, capital and/or habitus to the empirical study of dementia care. Cowdell (2010), for example, used the concept of habitus to look at the way nurses are socialised into ways of working with people with dementia, whilst Twigg and Buse (2013) focused on clothing as a neglected, but important part of how we perform our identity, linking clothing choices of people living with dementia to Bourdieu’s (1984) work ‘Distinction’. In work focusing on embodiment and dementia, Kontos (2004) drew on ethnographic data and the ideas of Kitwood and Bredin (1992) to demonstrate how people living with dementia engaged their embodied self-hood to interact with those around them through gesture, physical contact, appearance, movement and dance. Whilst healthcare as a field, and interactions between family carers and people with dementia have been explored, there are currently no papers applying Bourdieu’s conceptual framework to oral health and experiences of the mouth. It is this gap that this paper seeks to fill.

Research suggests that dementia has a negative impact on oral health. This is largely based on studies with dental professionals or secondary analysis of quantitative data. Very few studies look directly at the experiences and views of people living with dementia, or their carers (family or friends). In addition, most research focuses on nursing or care home populations. Rising rates of dementia amongst an ageing, community dwelling, population retaining their teeth into old age suggest that this focus should move to the community (Daly et al., 2017). People with dementia are susceptible to the full range of oral diseases (caries, periodontal, disease and their sequelae), the prevention of which depends on good daily self/assisted-care, underpinned by regular professional care (Chideka et al., 2015). The potential impact of dementia on oral health is thus compounded by the fact that the ability to perform daily self-care may be undermined by impaired cognitive functioning, and access to professional care in a community setting may not be straightforward (Newton et al., 2018).

Drawing on data from a qualitative study exploring the experiences of the mouth and oral health amongst people living with dementia and their carers, this paper draws on Bourdieu’s concepts of field and capital to contextualise the experiences of people with dementia and their carers. Within this framework this paper sets out to do two things: to show that oral health and dental care are often an absent presence in the field of dementia care research and to demonstrate the implications of this for negotiating oral health and oral healthcare once the need for oral healthcare is recognised.

## Theorising a biological assault on the lifeworld

Dementia has the potential to impact all aspects of the daily lives of the affected person and their families. This is a multifaceted effect which can be causally attributed to the sociobiological attributes of the disease but is biological, social and psychological in its impact. To begin to understand the factors at play within dementia, a theoretical means for charting the interrelated experiences, choices and actions that occur is needed. Bourdieu’s (1991, 1998) concept of ‘field’ along with economic, social and cultural ‘capital’ provide a framework for doing this.

The field, for Bourdieu, refers to a structured social arena or space, bounded in an experiential context, in which actors attend to the self and/or a given situation. Fields are generated in any circumstances where practices are performed. Fields usually have a hierarchy built into them with their own power relations, struggles for dominance and forms of capital. The power struggles within the field influence the actions of all those who enter it and prompt certain types of behaviour, influenced by the distribution and transaction of capital which may be field specific or cut across multiple fields (Bourdieu, 1991). Bourdieu likens the actions that take place within a field to a ‘game’. The structures and inherent power struggles represent the ‘rules of the game’ and those within the field are ‘choosing’ to play the game. Thus people living with dementia and their carers making choices about the support that they need are, at least on some level, aware of the competing narratives of biomedicine, the market and of the state and accept that they need to negotiate these in order to receive support. They are invested in the game (illusio), believe in, and at least partially, understand the game and its stakes (doxa), and agree that the game is worth playing (collusion) (Bourdieu, 2000).

Research using Bourdieu’s concept of the field often relates this space to a specific arena in order to examine how the structures or rules shape the actions of those within it. This may be a disease such as Batten disease (Scambler and Newton, 2011) or a social space in which choices related to health service use are made (Collyer et al., 2015). By envisaging dementia as a field with biologically determined parameters, we can explore the ways in which the field of dementia and all it entails impose on the objects, institutions, structures and agents which find themselves within it.

Further, while fields are generated in any circumstances where practices are performed – inherent within them is a structured system of social positions and power relations formed by the distribution and interplay of various forms of capital. An exploration of the capital controlled and accessed by people living with dementia and their carers would then allow us to explore the relationship between affected individuals and the field in which they find themselves, incorporating patient/service user/carer/professional power relations, family relations and all of the minutiae of daily life affected by the disease onset, process, prognosis and outcome. Bourdieu (1991) distinguishes different forms of capital which operate throughout fields. This paper focuses on economic, cultural and social capital:

- Economic capital: directly convertible to commodity forms (e.g. money) and may be institutionalised (e.g. welfare rights or pensions).
- Cultural capital: socially ‘legitimated’ knowledge and practices which in certain circumstances is convertible to economic capital and is institutionalised through social sanctions such as educational qualifications.
- Social capital: networks, affiliations, social obligations and quality of relations with others which in certain circumstances is convertible to economic capital; and is institutionalised through repeated social networks.

The ways in which capital are utilised in the field of dementia are explored through the experiences of people living with dementia and their families.

## Theoretical modelling of the empirical data: In the field of dementia

The data utilised came from a study undertaken to explore the experiences of people with a diagnosis of dementia and/or their carers, in relation to the mouth and oral health. Participants were recruited from the UK’s ‘Join Dementia Research’ (31,000 registered volunteers) and included people who lived in private residences; had a diagnosis of dementia or had been the family carer of someone with dementia for at least 6 months and had the capacity to consent to participate in the study. Capacity to consent was determined using the legal framework of the Mental Capacity Act 2005 (Department of Health, 2005) operating in England and Wales and written informed consent was obtained from all participants following this process (see Curtis et al., 2021). Purposive sampling (Ritchie et al., 2013; Saks and Allsop, 2012) was employed to select participants who met the inclusion criteria which included capacity to consent to take part in an interview. In total 17 participants were recruited and 12 semi-structured face to face interviews conducted with 7 people diagnosed with dementia (5 female and 2 male) and 10 carers (7 female and 3 male). Five interviews were conducted with a carer alone (P1–5), two with a person living with dementia alone (P1–2) and five jointly with a person living with dementia and their carer (P3–7). Some participants experiencing dementia received care from relatives and neighbours, while others received support from staff such as district/community nurses, home care workers and occupational therapists.

Each interviews lasted approximately 60 minutes and a flexible topic guide informed by the literature was used to guide discussion with member checking used throughout the interviews to ensure shared understanding of experiences and meanings. All interviews were recorded and transcribed verbatim, with all transcripts anonymised individually and geographically to ensure that participants could not be recognised. Thematic content analysis was undertaken following the process outlined by Green and Thorogood (2018) with the aim of achieving a ‘rigorous and systematic analysis of data that results in the development of concepts and categories that emerge from the words of informants, culminating in the development of explanatory models’ (Silverman, 2020). Data were coded line by line deductively and inductively using a broad-based thematic framework drawn from the literature and expanding it through the coding and analysis process. A random selection of transcripts was double-blind coded by two members of the research team and was used to enhance the trustworthiness of the analysis process. Ethical approval was in place for this study from King’s College London (HR-17/18-5364) including procedures of what to do if there were concerns about safeguarding of the participants and all participants gave written informed consent.

The lead researcher is a social anthropologist with experience and knowledge of ethnographic data collection and dementia care provision; she had no previous relationship with any of the participants. Given the potential ethnocentric interpretation that can occur when having previous experience of the research topic, the interviewer, thus, employed phenomenological principles and engaged with participants in a manner which highlighted their stories and experiences. The wider research team included experts in dementia care, sociology and public health dentistry and included former family carers.

## Defining the field

From symptom onset through the often-lengthy pre-diagnosis period to the confirmation of diagnosis and the start of the post-diagnostic process of reorientation, reconstitution and adjustment, dementia has a profound impact on the daily life of both the affected person and their family (Campbell et al., 2016). The impact of the disease moves beyond the multiple challenges of daily life to incorporate, often complex, biomedical knowledge-based activities and decision-making procedures. The daily lives of people living with dementia, and their carers, are irrevocably bound to biomedicine regardless of the lack of a cure. Further, the process of negotiating day to day life necessarily combines the social, emotional and biological consequences of living with a disease of this type and can be understood only as a bio-psycho-social interaction. The starting point for an analysis of the impact of dementia on daily life is the point of symptom onset. It is at this point that affected people enter the field of dementia. As suggested previously, the parameters of the field are biological and both temporal and temporary. Parameters change over time with the development of new knowledge, technologies and therapies, and the entire field only exists in its present form as long as there is no cure or reconceptualisation of the condition.

The field is entered through the formal process of diagnosis where affected people receive the requisite diagnostic label and receive the legitimation of their place within the field. The process of diagnosis is not always straightforward however and may take a period of months. Affected people and their families may linger on the periphery of the field, sometimes for extended periods of time post symptom onset, whilst awaiting the legitimation required to fully enter the field:

*Well we didn’t know what’s going on for some time and it was when my mum started to lose her things and then she thought someone was coming in and taking and then she started tying up the doors . . .She called the neighbours at 2 o’clock and said things have been taken can you call the police, so they called the police who came twice and they said they couldn’t find any sign of breakage or any sign of anyone forcing the door. . . we ended up in [hospital] and they referred us to the GP saying they need to investigate further and they need to write a letter to Mental Health team to check. We came back to [hospital] for the brain scan and some other things. . .we had people who were coming in like physiotherapist I think, who would give her simple tasks like hoovering and making tea and things and they would observe her. . . (Participant 14, Female, Daughter of a person with dementia, aged 42* *years)*

Because of the nature of the symptoms, it was often family members who first noticed changes, prompting investigations that led to diagnosis and entry to the field:

*I didn’t pick it up, my daughter and my ex-wife picked it up. They noticed little incidents of memory failure, about a year before we went to the doctor, in fact they pushed me into it, both of them, which was a good move. I found a Neurologist at [hospital] who’s rather good. (Participant 7, Male, person with dementia, aged 83* *years)*

Whilst the individual pathways are different, for many there is a shared experience of an extended diagnosis process involving a range of psychosocial and biomedical tests that result in a diagnosis and admittance to the field.

## Mapping the field of dementia

On gaining entry to the field, people with dementia and their carers find themselves in a structured social space. The field of dementia is structured by social class, gender, ethnicity and age, as are all social spaces, but also, by the health, social care and welfare systems that order and shape the space within the field. Each system incorporates organisations which provide, to some extent, care, services, information, support and equipment. This is the space in which people with dementia and their carers find themselves having received their diagnosis. While adjusting to a diagnostic label that often brings fear and potentially stigma, and to symptoms which both fluctuate and challenge the ability to deal with that fluctuation, they find themselves in a crowded social space filled with organisations and services that may be useful but often with no obvious road map for families to follow, and no way of telling where they need to go and how to get there. The overwhelming nature of this is illustrated in the quote below:

*Yeah, cos when she came to live with us, it was a case of dealing with everything head to toe. . .. So, both cataracts done very quickly, dentist, the eye test.. . . she hadn’t had her eyes done or checked even for ages . . .. . .I said, well we were led to believe mum was having carers [paid care workers] . . .She [care coordinator] said, ‘no. . .. No, there’s no money’. . ..She said, ‘we can’t’ . . . And I said, ‘well somebody should have . . .’. (P3, female, daughter of a person with dementia, aged 59* *years)*

A broad range of needs, exacerbated by a lack of co-ordination and financial constraints, can make caring overwhelming for families.

A lack of communication between various agencies, even when they are part of the same, albeit large, organisation such as the NHS, can make the situation even more difficult and overwhelming for both affected people and carers.

*. . .unfortunately what happens particularly in the NHS is one person does one job and they don’t link into anybody else and they come out and they do it and then the forget about it and there’s never a follow up to it. So, if someone gets issued with a hearing aid on the NHS that’s it, end of story, they’re discharged, . . .and there’s never any follow up to it. (P6, male, son of a person with dementia, aged 66* *years)*

And all of this occurs in a field which is also structured by the nature of the condition itself which can render even the limited communication that may be available unreliable due to memory loss and confusion. The frustration felt here is palpable and in taking over his mother’s care this participant (P6) found himself in what we might term a ‘non-doxic’ field. That is a social space where the ‘rules of the game’ or the ways in which the social space should be navigated and the resources necessary to do this, are unfamiliar. In this instance they are unfamiliar because of the bio-medicalised institutions which structure the field and because of the nature of the condition itself – specifically the ways in which it undermines agency. The non-doxic field of dementia can challenge carers’ ideas about their roles, their relationships with their partners or parents and what is required of them.

## Locating dental care within the field

Where does dentistry fit into this unfamiliar field? The accounts of participants suggest a range of barriers to accessing dental care within the field of dementia. These can be split into: access; attitudes towards oral health and the wider perception of ageing and disability. Barriers to access include physical difficulties accessing clinics, difficulties finding and arranging community-based dental care, associated problems related to access for carers and an overall lack of information on types of dental care available and how to contact the service. Often these barriers work in concert:

*I don’t have time to go for lots and lots of treatment and sort things out. . .We’re waiting for a wheelchair, the physio yesterday tried to hurry it up, but we’ve been told it’s going to be another two months. . .We’d only be able to get physically there in a taxi, [and] we would need to do it on a day that our daughter was around to help get him down the stairs. (P11, female, non-family carer)*

Multiple barriers are encountered here relating to equipment, negotiating with professionals, the cost of transport and the need to co-ordinate visits with the availability of their daughter so that she can assist. Each of these barriers need to be successfully negotiated in order to access primary dental care.

If community based domiciliary dental care is needed then a different set of barriers is encountered and accounts focused on a lack of information about the availability of domiciliary dental care and how to access it. Even when participants were able to secure a domiciliary visit, the dental care provided did not always meet expectations:

*The dentist from [community service] came. . ..and he said she’s got several broken teeth that we didn’t know about, they were at the back mainly. He said is it causing pain? and I said no, I don’t think so. . .. . .but I got the impression that there was nothing he was going to do anyway. . .. . .He just said well you know as long as she’s not in pain everything, these teeth are broken and there’s nothing we can really do, and it was, you know, if she is in pain I’ll do something but if she’s not we’ll just leave her. (P14, female, daughter of a person with dementia, aged 42* *years)*

This quote highlights two further barriers to accessing dental care within the field of dementia, those of ageism and ableism. Whilst many factors may affect the treating dentist’s decision-making process, ageism and ableism are perceived to shape the response of the dentist and the expectation that no treatment will be provided in this case unless there is pain. The carer recounting this story suggested that for younger, non-dementia, patients leaving broken teeth untreated would not be seen as acceptable.

In addition to these external barriers, there is also a lack of perceived need – or a low prioritisation of oral care, both from people with dementia and their carers, and also from other allied health professionals. One carer highlighted a lack of information or awareness of oral health as an issue amongst the health professionals she encountered:

*Yes, and he’s on a modified diet because of his swallow and I do, I mean I’m quite, not obsessive, but I’m very conscious of keeping him hydrated and mixing water in with everything else but on reflection the district nurses don’t mention it [oral health] either, you know they check the catheter, they check the [unclear] but they don’t think about the mouth. . .. when I think about it everyone worries about you know, getting chest infections, getting urinary infections, you could quite easily get an infection in your mouth couldn’t you? (P17, female, non-family carer)*

This ties in with the paucity of guidelines and recommendations on oral health at home highlighted in the introduction to this paper and suggests that dentistry, and oral health care more widely, can be seen as an ‘absent presence’ in the field of dementia.

Other participants highlighted oral health as simply one of many problems to be addressed on an ongoing basis. Both carers and people with dementia highlighted the difficulties encountered juggling multiple health needs:

*It feels like incredibly busy [during] the day trying to get it all [health requirements] in. I take lots of tablets and I’ve got to remember. . . I have my own technique for that. . . but if I don’t use my technique I don’t even remember [if I’ve taken] my tablets. . . I’ve got to really concentrate to keep the blood pressure [down]. . . and so that’s important, so I try and do it [take the tablets] at a set time and that takes precedence over teeth. (P12, female, person with dementia, aged 60* *yerrs)*

Or dealing with other competing priorities such as ensuring enough food is eaten:

*I would spoon feed her sugar if that’s all there was to keep her alive (P1, Female daughter of a person with dementia, aged 60* *years)*

And again, this was underpinned by the assumption that oral health becomes less of a priority with ageing:

*But do I need anything done to make my life better during the next 10 years? That’s my sort of. . .the way I look at it. . .. . . . . .as long as they [his teeth] see me out, that’s the only thing that matters. (P4, male, person with dementia, aged 87* *years)*

When locating oral healthcare within the field then, in line with the wider literature, this analysis suggests that dentistry is routinely excluded from the health and allied health institutions that help structure the field of dementia. Information is scarce and oral health is not part of the wider narratives around health and healthcare unless and until a specific issue arises. At that point, the difficulties accessing dental care become apparent. In addition, awareness of the need for dental care and oral health more widely is challenged by competing priorities and a perception that oral health is, and should be, a low priority for older people with dementia, both amongst people with dementia and their carers, but also potentially amongst the dental and other health care professionals encountered within the field. This leads to dentistry existing as an ‘absent presence’ in the field of dementia – only noticeable by its absence when people are actively seeking it. At this stage capital comes in to play as people within the field use the resources, they can mobilise, in the form of power, to position themselves within the field and access the care that they need.

## Capital accumulation and transactions

All three of the forms of capital outlined in the first part of the paper (economic, cultural and social) are utilised by people living with dementia or more often their carers negotiating their way through the field of dementia and, more specifically here, in accessing, or attempting to access dental care. The ability to carve out a positive life experience and forge the necessary relations within and across positions in the field to maintain oral health is dependent on capital. The ways in which people accumulate and use capital, and issues encountered where sufficient capital is not available, are addressed here.

### Economic capital

Economic capital can refer to the money or resources available to people with dementia and their carers to adapt to, live with and combat the condition and its symptoms. Economic capital may be accumulated in the form of personal wealth, benefits and/or state intervention in the form of subsidised or freely available services. State based economic capital is institutionalised through the health and welfare systems. Economic capital may take the form of equipment and services, the lack of which affects the accumulation and transaction of other types of capital and access to necessary services. One participant, for example, as highlighted above (P11), talked about waiting for a much-needed wheelchair to be provided by the NHS. In this case economic capital comes in the form of necessary equipment without which services cannot be accessed. Institutional economic capital (predominantly health and welfare) may mitigate against the loss of personal economic capital as in the case above. In some cases, institutional economic capital in the form of welfare (in this case a state pension) may not be sufficient to cover the cost of dental care where most UK adults have to make co-payments even for basic care:

*I’m on a very meagre pension and so I really can’t afford more than the cursory dental care. (P7, male, person with dementia, aged 83 years)*

In other cases, however, necessary services and equipment appear to be excluded from institutional economic provision, as in the case of dentures:

*Yeah, you send it away to the lab and they charge fees. Now of course privatise that, but I don’t need an exam, I need an urgent repair to get the teeth in the front attached to the back part of the plate for me to smile, be able to talk and smile. (P12 female, person with dementia, aged 60 years)*

This may leave those with insufficient economic capital unable to access the care they need. This can affect both carers and people with dementia:

*Because I know I go to see the dentist I’m going to have one, two, three, four thousand pounds worth of work. . .. I don’t have that money. I can’t afford to see a dentist, it’s as simple as that and you asked about granny’s wallies [dentures], who’s going to pay for granny’s wallies. I think somebody did mention them and it wasn’t a matter of they won’t be replaced, it was a matter of it will cost you some stupid money to have them replaced, stupid money we don’t have. (P16, Male son of a person with dementia, aged 64 years)*

Economic capital, in the form of money may mitigate against the loss of other forms of capital, being used to buy in dental services, for example, where family care is not available, but clearly a lack of economic capital makes this impossible. The cost of dental care was raised by a number of participants, however, and both those with and without access to economic capital were aware of the impact of not having sufficient resources on the ability to access care. For some this was the direct cost of accessing dental care:

*There’s also an issue though that some people don’t have the money to go and see the dentist, and that’s a real issue, isn’t it? (P8, female, person with dementia, aged 64 years)*

For others it was linked to access to wider support systems. One couple, for example, was able to use their resources to live in a private supported apartment within a complex which provided, for a fee, health and care on site along with hair and beauty grooming services and a range of other services. Furthermore, those with higher levels of economic capital were more likely to be able to purchase the domestic and personal care needed to stay within their homes, maintaining at least some perceived independence, whilst those without the resources were more reliant on family members to provide such care. That said, even those with significant economic capital often needed social capital to make use of their economic capital resources. Because of the nature of the condition, decreasing independence meant that money alone was not sufficient to ensure quality care. Thus, whilst economic capital is the most obvious form of capital to impact on access to, and use of, formal oral health care services in the field of dementia, cultural and social capital proved equally valuable in negotiating power and position in the field.

## Cultural capital

Cultural capital is based on socially ‘legitimated’ knowledge and practices which in certain circumstances is convertible to economic capital and is institutionalised through social sanctions such as educational qualifications. People entering the field of dementia may face a challenge to their cultural capital reserves and their ability to build more capital as they need to develop new knowledges and practices; specifically around the disease and its process including the ability to negotiate and organise complex packages of care and communicate with a range of health and allied health professionals within the field. The results of this analysis suggest that cultural capital is the key to successful negotiations with the multitude of professionals encountered within the field of dementia. This, combined with detailed knowledge of the person that care is being provided for, is essential in the management of a complex and often uncertain condition.

Some of the participants in this study were able to draw on expert cultural capital to help negotiate their way through the field, either directly or indirectly. One participant, for example, talked about using her nursing background to access information about available services:

*I was a community nurse . . . was my last job so yeah . . . and things like this Day Centre where she goes, we can . . . we get information through there as well. (P3 female, daughter of a person with dementia, aged 59 years)*

Another participant with dementia was aware of the expert capital that he had access to through his daughter – to better understand the relationship between his dementia and other areas such as oral health:

*There’s an association between dementia and oral health. Isn’t that right? Our elder daughter was a health journalist [ah hah]. She’s not anymore, but anyway, so, she’s aware of all these issues that can happen. (P4 male, person with dementia, aged 87 years)*

In both examples, participants brought into the field with them forms of cultural capital that could be used to better negotiate access to care. In most cases, however, family carers are neither experts in the professional sense of the term, nor expert patients or carers. Yet they are pivotal in providing care.

Those participants who did not have a form of expert cultural capital to draw on within the field often had other forms of capital that they could apply. One carer, for example, talked about the range of information sources that she knew were available and that she could contact to find out about services and how to access them:

*Well, we would, it may be, that we might need to find a dentist that does do home visits. I know you can always contact Age Concern and . . . . . . there are some companies . . . businesses, groups that do a domiciliary visit so that would be another option. (P3, female, daughter of a person with dementia, aged 59 years)*

Another talked about her plan to ‘schmooze’ the hygienist in an attempt to persuade her to pay an additional home visit to her mother:

*it sounds terrible but I’m going to schmooze her a bit because that’s what I did the first time. . . .. . . I talk to anybody and I think I’m quite good with people and she said oh I think we could do one more visit but then she explained the discharge and blah, blah, blah. So, I’m going to try and schmooze her again on Friday to see if she’ll come out one more time and one more time, but I shouldn’t have to do that, she maybe in a bad mood on Friday and say no that’s it. (P1, female, daughter of a person with dementia, aged 60 years)*

In this example the cultural capital comes in the form of the ability to communicate with people and persuade them to do what is needed. Whilst this is not a socially legitimated set of practices per se, it nevertheless represents cultural skills that are brought into the field and can be used as a means of trying to negotiate. All of the examples here demonstrate how people use a range of expert and non-expert cultural capital resources in attempts to negotiate their way through the field of dementia. Although all the participants in this study found themselves in the same, often disjointed, confusing, biomedicalised field, their ability to negotiate a position within the field was shaped significantly by the types of cultural capital that they were able to access.

## Social capital

The final form of capital identified within this study was social capital. This relates to the networks, affiliations, social obligations and quality of relations with others that people brought with them and were able to call on. This type of capital can be found in the formal and informal support networks available to and drawn on by affected people and their families and may be challenged by the loss or non-repetition of previous forms of support. Social capital is at the heart of positioning within this field and takes two forms. Informal social capital takes the form of family and friends whilst formal social capital is provided by the institutions of health and social care or by a mixture of businesses and charities. Different forms of social capital are used for different purposes. For example, formal social capital may take the form of paid care workers coming into the home daily to maintain some independence both for people with dementia and their carers:

*I have thought about mum coming here but to be honest she has such a good package at the moment, I have to go to work so I’m leaving her here and I couldn’t cope with it, it would drive me bonkers. . .. . .I’m finding it hard and I don’t live here. (P1, female, daughter of a person with dementia, aged 60 years)*

If, as in this example, people are happy with the quality of the formal social capital they can use, this can influence the ability of both the affected person and their families to live life in a way they would choose.

Informal social capital is most often seen in the provision of ongoing care by family members. This may involve engagement with the kind of negotiations highlighted in the previous section, but it can also involve hands on engagement with the day-to-day mundanity of life:

*My son, he helps. . .He prompts; he does prompt the medication, not so much the teeth. He prompts the teeth by doing it himself, so he walks by me with his electric toothbrush . . . (P12, female, person with dementia, aged 60 years)*

In this example, the son is not directly providing care, but is acting as a prompt to enable his mother to self-care. Other carers talked about providing meals, and personal care such as washing and dressing. In one interview a daughter talked about using social capital to provide a more direct form of oral health care when formal services could not be accessed:

*I haven’t had any contact with the NHS dental service with mum at all and then last year she broke a tooth, and I didn’t know where to go or who to contact. I got ferried around from pillar to post, forms to fill in, different people to call and it was all very hard, and it should’ve been simple and then I found out there was an incredibly long waiting list. . . So, it seems like the one, solitary mobile dentist who’s doing the whole of the borough [has] got a huge waiting list, in the meantime she’s got a broken tooth . . . I was quite shocked when that wasn’t deemed an emergency. [It was] rubbing her gum but that wasn’t considered enough of an emergency. . . So thankfully, it sounds terrible, but her brother had a file and he filed it down. . .and that took off the edge. . . (P1, female, daughter of a person with dementia, aged 60 years)*

Although this is clearly an extreme example, it demonstrates the range of activities enabled by those with access to social capital and how it can be used where cultural capital and economic capital resources are weak.

What these findings suggest is that social capital is at the heart of provision of care but works alongside cultural and economic capital. Ideally people with dementia need the right kind of social capital resources which itself comes with the right kind of cultural capital to provide optimal negotiation through the field of dementia. Economic capital then facilitates the journey making it easier to access the care needed, whilst a lack of economic, social and cultural capital makes the journey far more challenging and less likely to be successful.

## Discussion

Dementia impacts all aspects of daily life and involves complex interactions with multiple professional groups, institutions and organisations in the health, social care, charity and business sectors. Using oral health and access to dental care as the example, this paper has sought to show how a Bourdieusian framework can help illuminate some of the power structures at play within the field of dementia whilst focusing on how people who find themselves within this field negotiate their way through it. This analysis suggests some potential reasons why people with dementia are less likely to visit a dentist regularly than other groups and more likely to attend at later stages where symptoms are severe (Lee et al., 2015). The reported lack of oral healthcare provision within ‘packages’ of care, the difficulty finding out about and accessing community or domiciliary dental care, and the reliance on relatives with the knowledge, skills or resources to make the necessary links all contribute to the poor oral health and lack of access to care highlighted by Chideka et al. (2015). This is likely to become an increasingly significant focus for both research and future healthcare provision with growing numbers of people living with dementia and an ageing, community dwelling, population retaining their teeth into old age (Daly et al., 2017).

In her work on healthcare interactions and inequality in care, Shim (2010) developed the concept of cultural health capital. This is a set of specific skills, behaviours and styles of interaction that maximise the chances of patients having positive encounters with healthcare professionals and are recognised as valuable by patients and healthcare workers. When applying this concept to patient centred care, Dubbin et al. (2013) outlined the components of cultural health capital, which can include knowledge of medications and healthcare conditions, or the ability to communicate that knowledge clearly and efficiently, the ability to adjust interactional style and organisational skills. Significantly it also incorporates cues of favourable social and economic status. This form of specific cultural capital can clearly be seen in the stories of the people with dementia and carers interviewed in this study. Biomedical knowledge, organisational skills and interaction styles were all brought into play when trying to access care and maintain oral health but were heavily reliant on the type of social capital people with dementia were able to draw on. Those with carers who had their own cultural health capital were more able to negotiate access to care as needed.

As previously highlighted, a lack of access and perceived need for oral healthcare are compounded by the absence of clear guidance on the provision of oral health care for people with dementia. NICE guidelines on ‘Oral Health for Adults in Care’ offer little in the way of guidance (National Institute of Clinical Excellence, 2018) and Curtis et al. (2021) argue that better understanding of oral health needs and experiences of people with dementia and their carers, and of the dental services available to people with dementia, are needed. This research suggests that oral health is not a priority unless there is pain and then it comes to the fore. Furthermore, dental care, the majority of which is delivered in mixed private and NHS primary care settings, separate from other aspects of health and social care, is largely invisible as a specialist service in the field of dementia. People carry on accessing standard general dental care until they can no longer do so and then they stop accessing care altogether unless there is a crisis (Curtis et al., 2021). This may also be the case for some carers whose access is restricted by their caring roles. When specialist care is required there is a dearth of readily available information on the existence of community and/or domiciliary dental care and where or how to access it, (the absent presence of dental care in the field), underpinned by the lack (or lack of visibility) of community services and what could potentially be seen as underlying ageism and/or ableism as part of the structure of the field which presupposes low need in this patient population. For dementia care practitioners mention of oral health could be part of regular annual reviews that people living with dementia are entitled to from NHS primary care under the Quality and Outcome Framework (Dow et al., 2020). Other practitioners could be encouraged to consider oral health care more proactively and post-diagnostic consultations could also include mention of local services and how to access them. Carers may also find that peer-support groups can enhance their knowledge and confidence in accessing services and should be advised about such support along the illness trajectory. In England the new roles of social prescribers in primary care may assist both people living with dementia and carers in navigating the complexities of local services as illustrated above, but they will need to build up accurate pictures of local provision in order to link or bridge informal social capital to more formal forms of support.

The limitations of this study also need to be acknowledged. The research that this paper draws on was conducted in one area of England at one timepoint. This meant that we were not able to explore people’s views through the stages of dementia or in different areas of the country. In addition, because of the recruitment strategy participants are more likely to be from social groups that value research and have the time and interest to contribute. While it could be argued that the sample size is small, it is in line with other qualitative explorative studies of this type (Bissett et al., 2013; Dharamsi et al., 2010; O’Reilly and Parker, 2013) and enabled us to collect rich, nuanced participant accounts.

## Conclusion

This paper provides a new way of thinking about oral health and the provision of oral health care for people with dementia. It highlights the low priority of oral health until problems arise and difficulties faced accessing care when needed. Pathways are complex and information is scarce. An understanding of the resources that people draw on to negotiate care within the field is essential if we are to better organise and deliver effective oral health care to this growing, vulnerable section of the population and to enable them to challenge the inequities described by participants in this study.
